# Healthcare use and costs of perinatal anxiety: a UK NHS perspective

**DOI:** 10.1186/s12913-025-13318-z

**Published:** 2025-09-03

**Authors:** Kalpa Pisavadia, Kodchawan Doungsong, Victory Ezeofor, Llinos Haf Spencer, Lorna Tuersley, Catherine Best, Rose Meades, Susan Ayers, Una Hutton, Patricia M. Moran, Judy Shakespeare, Andrea Sinesi, Rhiannon Tudor Edwards, the MAP ALLIANCE study team, Margaret Maxwell, Helen Cheyne, Kathryn Hollins, Fiona Alderdice, Amy Delicate, Jennifer Holly, Rafiyah Khan, Rachel Leonard, Debra Salmon, Lily Strange

**Affiliations:** 1 Centre for Health Economics and Medicines Evaluation (CHEME), Bangor University, Bangor, Gwynedd, Wales, LL57 2PZ UK; 2 Faculty of Life Sciences and Education, Health, Care, and Well-Being Research and Innovation Group, University of South Wales, Pontypridd, Wales, CF37 4BD UK; 3 Faculty of Health Science and Sport, University of Stirling, Stirling, Scotland, FK9 4LA UK; 4 Centre for Maternal and Child Health Research, City, University of London, Northampton Square, London, England, EC1V 0HB UK; 5 Retired General Practitioner (GP), Oxford, England, UK

**Keywords:** Perinatal anxiety, Women, Birth, Pregnancy, Healthcare resource use, Health services, Cost of illness, Health economics

## Abstract

**Background:**

Perinatal anxiety is a significant public health issue with potential adverse outcomes for both mothers and their babies. This study provides a comprehensive analysis of the costs associated with health service use for women with and without perinatal anxiety in the UK National Health Service (NHS) at six and twelve months postpartum. This research is part of the MAP Alliance Study—a large programme of research on perinatal anxiety.

**Methods:**

A cost of illness (COI) analysis was performed using a retrospective approach in which recorded data of self-reported health resource use was analysed. The COI analysis identified the different components of costs and the size of the contribution of each health resource and quantified the direct costs incurred by the NHS due to perinatal anxiety.

**Results:**

Results found that women experiencing perinatal anxiety use healthcare services more frequently and incur higher healthcare costs compared to those without. At six months postpartum, the mean total healthcare cost per woman with perinatal anxiety was £1174 (95% CI: 1080.67, 1263.05) compared with £1046 (95% CI: 975.16, 1123.83) for women without. At twelve months postpartum, the mean total healthcare cost per woman with perinatal anxiety was £414 (95% CI: 347.76, 488.87) compared with £267 (95% CI: 226.06, 314.81) for women without. However, this cost difference between the two groups was not statistically significant (-£14; 95% CI: -161.88, 135.65, *p* = 0.808).

**Conclusion:**

These findings underscore the economic impact of perinatal anxiety on healthcare systems and highlight the need for targeted interventions to improve care pathways for affected women. The results of this analysis have significant implications for public health policy, emphasising the importance of optimising perinatal mental health care pathways to reduce long-term costs and improve outcomes for women.

**Supplementary Information:**

The online version contains supplementary material available at 10.1186/s12913-025-13318-z.

## Introduction

Perinatal mental health (PMH) problems are among the most common complications during pregnancy and the postpartum period, affecting one in five women [1]. Perinatal anxiety, a significant but under-researched aspect of PMH, has far-reaching consequences for women, their families, and society. Without effective treatment, perinatal anxiety can lead to adverse maternal and child health outcomes, including preterm birth, postnatal depression, and developmental challenges for children [2–4].

In the UK, the societal cost of perinatal mental health problems is estimated at £8.1 billion annually, with 72% of this cost attributed to the long-term impact on children [5]. While extensive research has been conducted on postnatal depression, the economic burden of perinatal anxiety remains largely unexplored [6]. Addressing this gap is crucial for understanding the financial and healthcare implications of perinatal anxiety and for optimising service delivery.

Government and healthcare initiatives in the UK recognise the importance of PMH, with significant investment and commitment to improving perinatal mental health services across the UK. Initiatives include NHS England’s 2016 commitment to nationwide specialist perinatal community services and Scotland’s £52 million investment in perinatal and infant mental health services represent progress toward improved care [7, 8]. However, there is limited research on how women with perinatal anxiety utilise these services and whether these investments have translated into cost-effective and accessible care.

This study addresses these critical gaps by analysing the cost of illness (COI) associated with perinatal anxiety. Through the MAP ALLIANCE study, a longitudinal cohort tracking women with and without perinatal anxiety, this research evaluates healthcare resource use and associated costs at six and twelve months postpartum. Findings from this study will provide valuable insights into service use patterns, disparities in care, and the economic implications of perinatal anxiety.

This research aligns with international priorities, such as the World Health Organization’s guidelines on integrating PMH care into maternal and child health services to improve early childhood development outcomes [9]. It also contributes to the evaluation of current clinical policies and strategies aimed at enhancing PMH care accessibility and effectiveness in the UK [8, 10].

By generating evidence on the healthcare and economic burden of perinatal anxiety, this study aims to inform policymakers and healthcare providers about the progress and impact of PMH initiatives. The findings will support the development of targeted, culturally appropriate, and cost-effective interventions to reduce the burden of perinatal anxiety on women, their families, and the NHS, ultimately improving health outcomes and reducing avoidable costs.

## Materials and methods

### Setting and perspective

#### MAP cohort sample characteristics

The MAP cohort was a systematic sample of 2,243 pregnant women recruited in 29 NHS sites from 12 NHS Trusts in England and 5 Health Boards in Scotland. Recruitment was conducted from November 2020 to November 2021. Women were eligible for inclusion in the MAP study if they met the following inclusion criteria: (i) aged 16 years or over; (ii) less than 15 weeks pregnant at the time of recruitment to enable longitudinal follow-up of mental health and healthcare use across the perinatal period; (iii) able to provide written informed consent to take part in the study, and (iv) with a level of English sufficient to understand and complete questionnaires in lay language. Both singleton and multiple pregnancies were eligible for inclusion.

The sample was recruited from Scotland (10%) and England (90%) to reflect the relative populations and number of births in the two nations. The cohort is representative and diverse: the sample in Scotland is representative of the general population [11], whereas the sample in England has greater diversity with a greater proportion of participants from Asian, Black African/Caribbean, mixed ethnicity or other non-white groups compared to the 2021 Census [11]. The MAP study included measures of anxiety, depression and general psychological distress in early pregnancy (mean = 11.4 weeks, SD = 2.0), mid-pregnancy (mean = 23.0 weeks, SD = 1.3), late pregnancy (mean = 31.9 weeks, SD = 1.2), and postpartum (mean = 7.9 weeks, SD = 2.4).

#### MAP ALLIANCE sample characteristics

One thousand nine hundred sixty-four women from the MAP study were eligible to participate in the MAP ALLIANCE study (1,742 from England and 222 from Scotland). All the women had indicated that they could be contacted for follow-up research. Recruitment was carried out between June 2022 and January 2023. Ethical approval required women to re-consent to take part in the MAP ALLIANCE study. Overall, 794 women consented to participate in the MAP ALLIANCE study. During timepoint one (0–6 months) and timepoint two (6–12 months), 1.2% (10/794) of participants withdrew and 0.5% (4/794) of participants discontinued. 722 women consented at six months and an additional 72 women consented at twelve months. Demographic and health variables such as age, ethnic group and experience of mental health problems were collected from participants at the early pregnancy time point as part of the MAP study.

### Perinatal anxiety assessment

The number of women with perinatal anxiety, six weeks postpartum, was determined by those who scored 9 or above on the Stirling Antenatal Anxiety Scale (SAAS) 10-item screening tool [12, 13]. The SAAS outcome tool was found to be an accurate diagnostic measure with high sensitivity to measure anxiety in a perinatal population. This measure has been found to be clinically relevant and psychometrically valid, with good diagnostic accuracy [13]. Scores were imputed using the Traj-mean Single imputation approach for participants with missing SAAS scores [14].

### Health resource use

Health service resource use was measured using items from an adapted Client Service Receipt Inventory (CSRI) questionnaire from birth to six months and then six to twelve months postpartum [15, 16]. Items from the CSRI were used to measure the health resource use of primary and secondary care for mothers and babies. Primary care resource use is comprised of contacts with the General Practitioner (GP), health visitor and community midwife. Secondary care resource use consisted of inpatient hospital stays, hospital services, and contact with other healthcare professionals (e.g. physiotherapists), as self-reported by participants. Participants with missing data on resource use were not imputed.

### Wider societal impact

The frequency of intention to return to work for those who did and did not return to work is presented to identify the wider societal impact of perinatal anxiety.

#### Cost of illness analysis

COI analysis is considered an essential healthcare evaluation technique to measure and compare the economic burden of diseases to society. A COI analysis can provide policymakers, healthcare providers, and researchers with a comprehensive understanding of the financial impact of a disease. By assessing the economic burden, decision-makers can make informed choices regarding resource allocation, healthcare planning, and policy development [17, 18].

COI analysis was conducted using a bottom-up approach in which the cost of services was based on the resource consumption of individual participants [19]. Due to the availability of a rich dataset, the bottom-up method, as opposed to the top-down approach, has proven to provide a more accurate capture of the health resources used by women with and without perinatal anxiety [20].

Although the MAP ALLIANCE study is a longitudinal follow-up of the MAP study, the COI component of the MAP ALLIANCE study was performed using a retrospective approach in which recorded data of self-reported health resource use was analysed. The COI analysis identified the different components of costs and the size of the contribution of each health resource and quantified the direct costs incurred by the National Health Service (NHS) due to perinatal anxiety. Resources used were multiplied by their unit costs in 2021/22 British pound sterling (GBP). Unit costs were collated from the Personal Social Service Research Unit (cost year 2022) and the National Cost Collection for the NHS (cost year 2021) [21, 22]. This mean cost was adjusted to the total number of participants receiving each service. For inpatient hospital stays, the reason for the stay was matched with the Health Resource Group description. An excess bed day charge was applied if the stay was longer than the trim point (calculated as the upper quartile length of stay for that Health Resource Group plus 1.5 times the inter-quartile range of length of stay) [23]. Due to a high range of costs attributed to the complexities of labour and delivery, the hospital inpatient labour cost has been excluded from the inpatient cost analysis. For hospital services and other healthcare professional visits, participants provided answers in free texts. The unit cost detail for health resource use is provided in Supplementary File 1.

The mean costs of each primary and secondary care service use are presented to the nearest pound sterling (£). Mean cost per patient at six months and twelve months postpartum are reported with 95% confidence intervals (CI) estimated using nonparametric bootstrap sampling. Five thousand samples were taken for each CI and bias corrected and accelerated confidence intervals were calculated. A nonparametric independent Mann–Whitney U test was performed to compare the difference between groups for the total healthcare costs between women with and without perinatal anxiety at twelve months postpartum. A *p*-value of less than 0.05 was set as statistical significance. A chi-square was performed to compare the difference in highest education level, parity, and previously experienced psychological/mental health problems. For marital status, ethnicity, parity (number of births after 20 weeks of gestation), and previous miscarriage or stillbirth, a Fisher’s Exact statistical test was used.

The frequency and distribution of primary and secondary service use are reported. All significant costs impacting health resource use were examined. Linear regression analysis was conducted to investigate significant predictors of costs [24]. For example, age, hospitalisation, ethnicity, Stirling Antenatal Anxiety Scale (SAAS) score and parity at both time points.

### Subgroup analysis

Ethnicity, parity, regional variation and adverse events such as miscarriage and stillbirth were examined for subgroup analysis to compare the difference within the mean of total healthcare resource use costs at six and twelve months. A large proportion of the sample came from England, and the regions were grouped as follows: North England, South England, London, Midlands and Scotland.

### Sensitivity analysis

Sensitivity analysis was conducted for twelve-month data that included an additional 72 women who only participated in twelve-month data collection. The interquartile range (IQR) was used to identify the outliers, which were then removed to explore the uncertainty of the result with and without outliers. Any costs that were less than Quartile 1-(1.5*IQR) or more than Quartile 3 + (1.5*IQR) were identified as outliers.

## Results

### Baseline characteristics

At six weeks, the number of women with perinatal anxiety was 274 and the number of women without perinatal anxiety was 448. The mean age of women with and without perinatal anxiety at baseline was similar (31 and 32 years, respectively). Women with and without perinatal anxiety differed in parity (*p* = 0.012), previously experienced psychological/mental health problems (*p* < 0.001), The baseline characteristics are summarised in Table 1.

**Table 1:** Baseline characteristics of participants at six months postpartum (*n* = 722)^a^

Women with perinatal anxiety (*n* = 274)	**With perinatal anxiety**	**Without perinatal anxiety**	***P*-value**
Women without perinatal anxiety (*n* = 448)	n (%)	n (%)	
Mean age, years (SD)	31 (4.66)	32 (5.52)	0.778
Marital status			0.295
Married/civil partnership	146 (56%)	255 (60%)	
Cohabitating	94 (36%)	139 (33%)	
In relationship, non-cohabiting	17 (6%)	20 (5%)	
Separated/divorced/single	2 (1%)	9 (2%)	
Prefer not to say	2 (1%)	1 (0%)	
Highest education level			0.185
None	3 (1%)	5 (1%)	
Secondary education	15 (6%)	27 (6%)	
Post-secondary education	33 (13%)	53 (13%)	
Vocational qualification	41 (15%)	36 (8%)	
Bachelor’s degree or equivalent	110 (42%)	200 (48%)	
Master’s degree or equivalent	48 (19%)	80 (19%)	
Doctorate	10 (4%)	20 (5%)	
Ethnicity			0.230
White British	231 (84%)	349 (78%)	
White Irish	2 (1%)	1 (0%)	
Asian	16 (6%)	34 (8%)	
Arab	0 (0%)	6 (1%)	
Black/Caribbean/Arab	2 (1%)	17 (4%)	
Mixed ethnicity	7 (3%)	15 (3%)	
Other ethnicity	2 (1%)	1 (0%)	
Missing data	14 (5%)	25 (6%)	
Parity			0.140
0	170 (62%)	195 (44%)	
≥ 1	104 (38%)	253 (56%)	
Previously had miscarriage or stillbirth			0.659
Yes	77 (52%)	119 (48%)	
No	69 (47%)	128 (51%)	
Prefer not to say	2 (1%)	3 (1%)	
Missing data	126 (46%)	198 (46%)	
Previously experienced psychological/mental health problems			< 0.001^*^
Yes	154 (58%)	102 (23%)	
No	94 (35%)	312 (72%)	
Don’t know	18 (7%)	22 (5%)	
Missing data	8 (3%)	12 (3%)	

^*^P value significant level at 0.05

^a^Missing data were not included in statistical analysis

### Health resource use

Primary and secondary care utilisation within six and twelve months postpartum are presented in Table 2. Women with perinatal anxiety had higher health service resource use than women without perinatal anxiety at six and twelve months postpartum. However, the duration of hospital stay was marginally higher in women without perinatal anxiety than women with perinatal anxiety at both time points.

**Table 2:** Mean number of healthcare service contacts for women with and without perinatal anxiety at six and twelve months postpartum

**Resource use category**	**6 months postpartum** n1,2 = 266, 438	**12 months postpartum** n1,2 = 201, 373
Primary healthcare service use		
Health visitor		
With perinatal anxiety	4.1 (0–20)	1.5 (0–10)
Without perinatal anxiety	3.5 (0–20)	1.2 (0–10)
Community midwife		
With perinatal anxiety	2.9 (0–14)	No data collection
Without perinatal anxiety	2.6 (0–10)	No data collection
GP		
With perinatal anxiety	1.8 (0–6)	1.3 (0–6)
Without perinatal anxiety	1.4 (0–6)	0.8 (0–6)
Secondary healthcare service use		
Hospital services		
With perinatal anxiety	0.5 (0–11)	0.6 (0–22)
Without perinatal anxiety	0.3 (0–10)	0.2 (0–11)
Hospital admission		
Number of admissions		
With perinatal anxiety	0.22 (0–2)	0.03 (0–1)
Without perinatal anxiety	0.23 (0–3)	0.02 (0–1)
Length of each admission (days)		
With perinatal anxiety	2.8 (0–9)	0.04 (0–4)
Without perinatal anxiety	2.8 (0–28)	0.06 (0–6)
Other healthcare professional (visits)		
With perinatal anxiety	2.1 (0–5)	1.0 (0–10)
Without perinatal anxiety	1.7 (0–5)	0.3 (0–5)

Values are mean (range)

n_1=_ number of women with perinatal anxiety; n_2=_ number of women without perinatal anxiety

No data collection indicates that previous employment status and intention to return to work were recorded at 6 months and were not re-collected at 12 months. At 12 months, only actual return or start of work was recorded

At six months postpartum, consultations with health visitors were the highest health resource used within primary healthcare, which varied between 0 and 20 visits for both groups. The mean health visitor resource use was 4.1 visits in women with perinatal anxiety and 3.5 visits in women without perinatal anxiety. For secondary healthcare, the mean length of stay for each hospital admission was three days for both groups. Women with perinatal anxiety used Accident and Emergency (A&E) (19% of services provided by hospitals) and midwifery services (19% of all services provided by hospitals). The outpatient department accounted for 23% of total hospital services for women without perinatal anxiety. For the category of ‘other health professionals’, physiotherapists were the most frequently consulted by both groups, which accounted for 20% and 30% of total other health professional visits for women with and without perinatal anxiety, respectively. Contact with mental health professionals for women with perinatal anxiety was 11% more for mental health nurses and 15% more for psychologists than women without perinatal anxiety.

At twelve months postpartum, consultations with health visitors were still the highest health resource use in primary healthcare. The mean health visitor resource use was reduced to 1.5 visits for women with and 1.2 visits for women without perinatal anxiety from six months. A&E and physiotherapy services had the highest proportion of hospital service use in women with and without perinatal anxiety (33% vs 27%, 17% vs 18%). The most healthcare professionals use amongst women with perinatal anxiety were psychologists (13%) and medical consultants (13%). The most healthcare professional use for women without perinatal anxiety were physiotherapists (35%) and medical consultants (13%).

### Primary and secondary healthcare costs

**Fig. 1:**
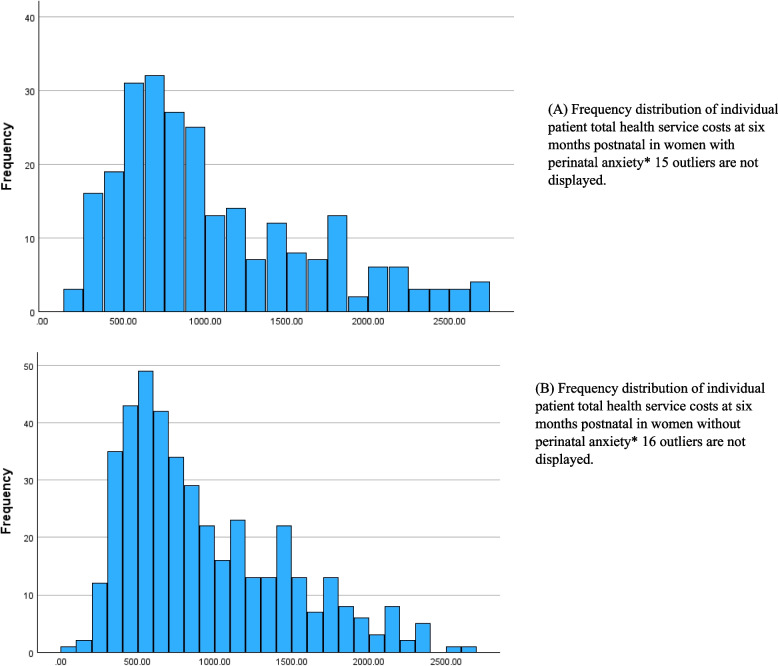
Frequency distribution of individual patient total health service costs at six months postnatal in women with and without perinatal anxiety. **A** Frequency distribution of individual patient total health service costs at six months postnatal in women with perinatal anxiety* 15 outliers are not displayed. **B** Frequency distribution of individual patient total health service costs at six months postnatal in women without perinatal anxiety* 16 outliers are not displayed

**Fig. 2:**
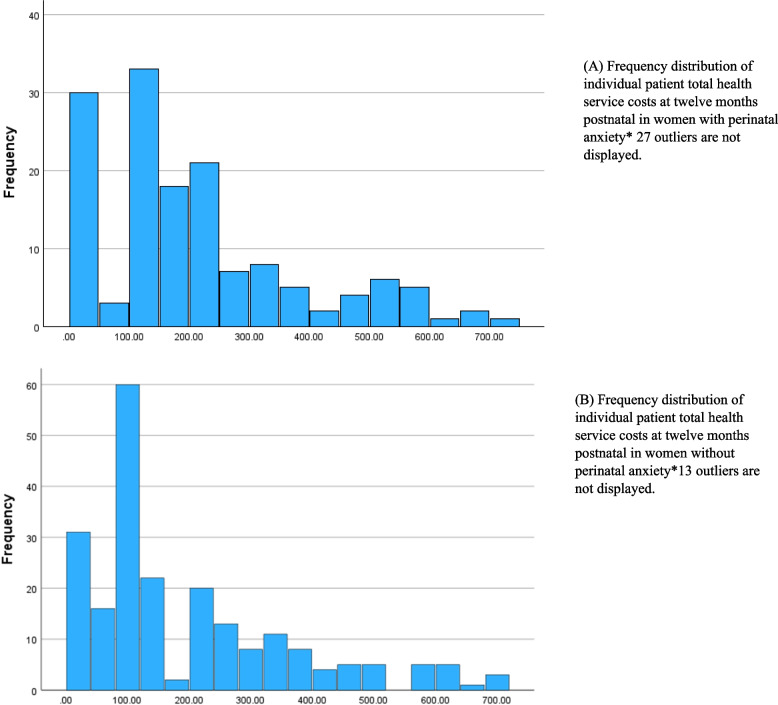
Frequency distribution of individual patient total health service costs at twelve months postnatal in women with and without perinatal anxiety. **A** Frequency distribution of individual patient total health service costs at twelve months postnatal in women with perinatal anxiety* 27 outliers are not displayed. **B** Frequency distribution of individual patient total health service costs at twelve months postnatal in women without perinatal anxiety*13 outliers are not displayed

Mean costs and 95% CI were calculated for all participants. The mean cost difference between six and twelve months postpartum and the mean change at twelve months between groups were calculated. Figures 1 and 2 provide a frequency distribution for individual participant costs and demonstrate highly skewed profiles. Six-month total healthcare costs ranged from £0 to £2500 while twelve-month total healthcare costs ranged from £0 to £800 for both groups.

**Fig. 3:**
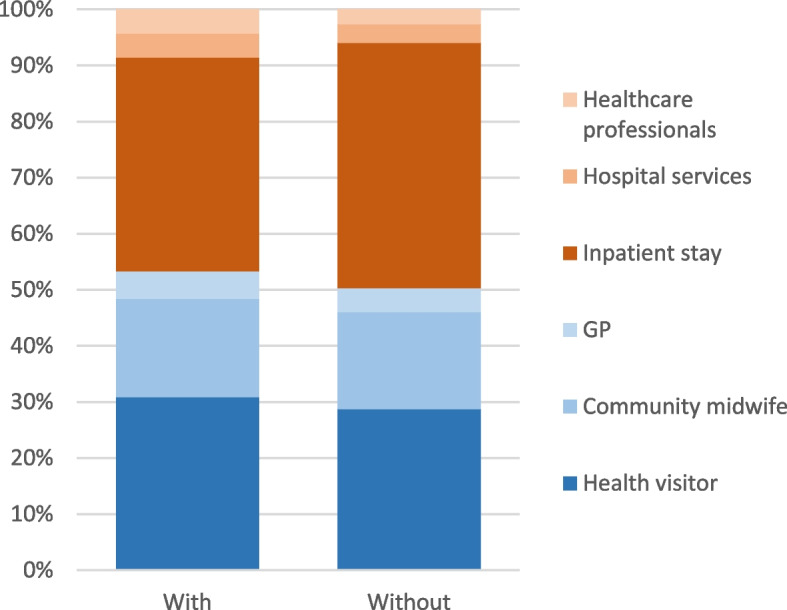
Contribution of different services to six month of total health care costs postnatal for women with and without perinatal anxiety (as per cent of the mean of totals health care costs). Primary health care and secondary health care are coloured in blue and orange themes, respectively

At six months postpartum, the mean of total healthcare costs for women with perinatal anxiety was £1174 [1080.67,1263.05], which is higher than for women without perinatal anxiety £1046 [975.16,1123.83]. Contact with health visitors accounted for 60% and 59% of total primary healthcare costs for women with and without perinatal anxiety, respectively (Table 3). Inpatient stay was the most significant contributor to secondary healthcare costs for both groups (women with anxiety £592; women without anxiety £619). Overall, secondary healthcare costs (inpatient stays) accounted for around 45% of the total healthcare costs at six months postpartum for both groups (Fig. 3).

**Table 3:** Mean costs of health resource use at six and twelve months postpartum (£, [95% CI])

Resource use category	6 months postpartum	12 months postpartum	Mean cost difference between 6 and 12 months postpartum	Comparison of total costs at 6 and 12 months between groups (With-without)	*P* value
Primary healthcare	n_1,2_ = 266, 438	n_1,2_ = 201, 368			
Health visitor				£27 [−24.08,77.64]	0.285
With	£480 [439.48, 523.76]	£179 [154.67, 204.21]	-£294 [−343.48, −246.40]		
Without	£407 [381.84, 432.25]	£141 [126.24, 157.24]	-£267 [−295.23, −240.35]		
Community midwife					
With	£271 [246.29, 295.77]	No data collection	NA	NA	NA
Without	£245 [230.20, 260.63]	No data collection			
GP				£2 [−11.19,15.68]	0.670
With	£78 [70.05, 85.36]	£54 [45.39, 63.11]	-£27 [−38.24, −16.02]		
Without	£60 [54.76, 64.68]	£35 [30.44, 39.94]	-£25 [−31.14, −18.91]		
Total costs of primary healthcare				£52 [−11.27,117.68]	0.065
With	£804 [757.58, 850.10]	£232 [205.31, 259.88]	-£573 [−630.55, −518.50]		
Without	£691 [658.03, 724.34]	£174 [157.88, 189.95]	-£521 [−556.45, −487.66]		
Secondary healthcare					
Inpatient stay					
With	£592 [454.04,740.83]	£49 [9.27, 104.51]	-£558 [−717.36, −408.80]	-£31 [−176.78, 250.03]	0.966
Without	£619 [498.52,754.29]	£43 [12.26, 79.91]	-£589 [−759.09, −449.94]		
Other hospital services				-£24 [−60.34, 12.88]	0.264
With	£66 [47.89, 86.72]	£71 [47.92, 96.14]	-£1 [−22.69, 24.39]		
Without	£48 [34.13, 63.72]	£30 [19.81, 42.26]	-£23 [−44.30, −3.73]		
Other healthcare professionals				£25 [−58.34, 8.84]	0.055
With	£67 [50.28, 85.45]	£82 [57.66, 109.02]	£9 [−23.52, 42.15]		
Without	£37 [28.68, 47.35]	£24 [17.50, 32.03]	-£16 [−28.64, −3.46]		
Total costs of secondary healthcare				£30 [−116.60, 180.85]	0.667
With	£431 [347.64,524.36]	£193 [134.04, 263.35]	-£257 [−376.85, −136.73]		
Without	£391 [322.90, 464.51]	£96 [60.37, 139.28]	-£287 [−382.68, −200.06]		
Total costs of healthcare				-£14 [−161.88, 135.65]	0.808
With	£1,174 [1,080.67,1,263.05]	£414 [347.76, 488.87]	-£791 [−910.88, −668.11]		
Without	£1,046 [975.16,1,123.83]	£267 [226.06, 314.81]	-£776 [−876.21, −682.83]		
Total costs of healthcare (outliers were removed)				-£66 [−190.91, 60.03]	0.237
With	£1,046 [975.81, 1,089.55]^*^	£204 [177.78, 231.02]^**^	-£752 [−850.49, −662.20]		
Without	£938 [889.07, 986.95]^*^	£199 [176.80, 222.08]^**^	-£687 [−764.53, −611.59]		

Costs are in £ in 2022 prices

*CI* confidence interval, *n*_*1,2*_ number of women with and without perinatal anxiety, respectively

^*^15 and 16 outliers were removed for women with and without perinatal anxiety, respectively

^**^27 and 13 outliers were removed for women with and without perinatal anxiety, respectively

**Fig. 4:**
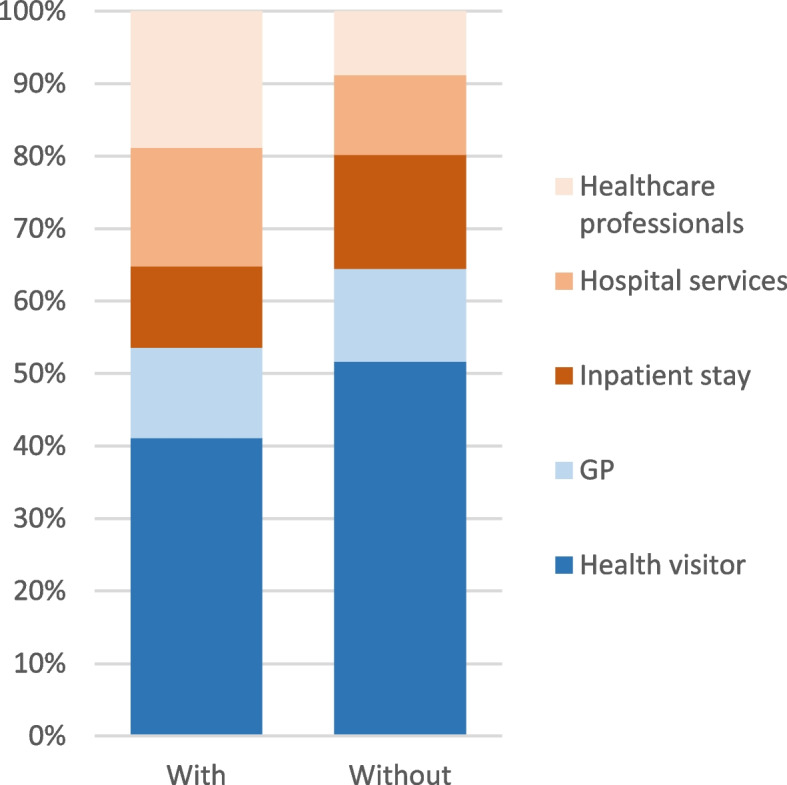
Contribution of different services to twelve month of total healthcare costs postnatal for women with and without perinatal anxiety (as a percentage of the mean of total health care costs). Primary health care and secondary health care are coloured in blue and orange themes, respectively

At twelve months postpartum the mean total healthcare costs for women with perinatal anxiety decreased to £414 [347.76, 488.87]. For women without perinatal anxiety, the mean total healthcare cost decreased to £267 [226.06, 314.81]. Health visitor visits were still the largest contributor to primary healthcare costs (Table 3). Hospital services and other healthcare professional visits significantly contributed to secondary healthcare costs for both groups. Interestingly, although secondary healthcare was the most significant contributor to total healthcare at six months postpartum, primary healthcare costs (health visitor) accounted for approximately 55%−60% of the total healthcare costs at twelve months postpartum for both groups (Fig. 4).

The mean total healthcare costs decreased by around £780 after twelve months postpartum for both groups due to greatly reduced inpatient stay costs (Table 3). There were no significant differences in health resource use costs between groups at twelve months (*p* = 0.808) (Table 3).

Sensitivity analysis confirmed that the mean total healthcare costs of women with perinatal anxiety was slightly higher than women without perinatal anxiety at both time points. At baseline, after removing outliers, the mean of total healthcare costs of women with and without anxiety were similar (women with perinatal anxiety ranged from £1174 to £1046; women without perinatal anxiety ranged from £1046 to £938). At twelve months, after removing outliers, the mean total healthcare costs of women with and without anxiety was similar (women with anxiety ranged from £252 to £265; women without perinatal anxiety ranged from £203 to £205). The pattern of healthcare resource use and the mean cost of healthcare resource use was similar regardless of whether the whole sample was used or if it was restricted to the 722 women who completed measures at both time points (see supplementary file).

Ethnicity, parity, regional variation and adverse events such as miscarriage and stillbirth history were investigated in subgroup analysis. At six months, there were higher mean total healthcare costs, regardless of perinatal anxiety status, for women who had any of the following characteristics: women who were of white ethnicity; their current baby was their first child; had experienced previous miscarriage or stillbirth; or lived in North England (see Supplementary file). At twelve months, the results of the mean total healthcare costs were inconsistent and varied between subgroups. Higher total healthcare costs were found for women with perinatal anxiety who were of white ethnicity, had been pregnant previously, and had experienced a previous miscarriage or stillbirth. The mean total healthcare costs of women with perinatal anxiety who lived in the Midlands was the highest among regions (£468). Within women without perinatal anxiety, the mean total healthcare cost was marginally higher for women who were of non-white ethnicity, had been pregnant previously, and had no history of miscarriage or stillbirth. Due to the small sample sizes of subgroup analysis results should be interpreted with caution.

Multivariate linear regression was used to assess the influence of age, hospitalisation, ethnicity, severity of anxiety (measured using the SAAS score) and parity in predicting total healthcare costs of women with and without perinatal anxiety who completed both six and twelve months postpartum (Table 4). Of these, only hospitalisation was significant predictor of the total healthcare costs at six months (*R*^2^ = 0.379, adjusted *R*^2^ = 0.373). In addition, age, the severity of anxiety, and hospitalisation were significant predictors of total healthcare costs at twelve months (*R*^2^ = 0.523, adjusted *R*^2^ = 0.521). There was a positive corelation between the severity of anxiety and total healthcare cost. For example, if the severity of anxiety increased by 1, the total healthcare cost at 12 months postpartum increased by £8.48 when age and hospitalisation are held constant. However, there was a negative correlation between age and total healthcare cost for every 1 unit increase in age, 12 months costs decrease by £8.49 when severity of anxiety and hospitalisation are held constant.

**Table 4:** Multivariate linear regression: influence age, parity, ethnicity, SAAS score, and hospitalisation on total 6- and 12-months healthcare resource use for women with and without perinatal anxiety

Variable	Coefficient (SE)	*P* value
6 months postpartum		
Hospitalisation	2,217.18 (122.45)	< 0.001
Constant	1,132.69 (401.17)	
12 months postpartum		
Severity of anxiety (determined by SAAs score)	8.48 (2.04)	< 0.001
Age	−8.49 (2.87)	0.003
Hospitalisation	2,408.28 (102.87)	< 0.001
Constant	475.58 (95.83)	

### Survey of intention to return to work and status of those who returned or started work

At six months postpartum, there was a same proportion of women with and without perinatal anxiety had previously employed. Questionnaires regarding intention to return to work and whether the participants actually returned or started to work were completed by 574 participants (80%) at six months and 533 (74%) at twelve months. At six months, a similar proportion of women with (80%) and without perinatal anxiety (82%) intended to return to work. Very few women had returned to work at six months (~ 10%) but by twelve months postpartum the percentage of women who had returned to work was slightly greater for women without perinatal anxiety (65%) than those with perinatal anxiety (60%) (Table 5).

**Table 5:** Intention to return to work and actual return or start of work at 6 and 12 months postpartum

Detail	6 months (*n* = 722)		12 months (who completed both 6 and 12 months) (*n* = 722)	
Previously employed	With (*n* = 205)	Without (*n* = 372)	No data collection	
Yes	187 (91%)	338 (91%)		
No	18 (9%)	34 (9%)		
Intention to return to work	With (*n* = 200)	Without (*n* = 369)	No data collection	
Yes	160 (80%)	302 (82%)		
No	40 (20%)	67 (18%)		
Returned or started work	With (*n* = 202)	Without (*n* = 372)	With (*n* = 183)	Without (*n* = 350)
Yes	20 (10%)	37 (10%)	109 (60%)	229 (65%)
No	182 (90%)	335 (90%)	74 (40%)	121 (35%)

No data collection indicates that previous employment status and intention to return to work were recorded at 6 months and were not re-collected at 12 months. At 12 months, only actual return or start of work was recorded

## Discussion

This study provides a comprehensive view of healthcare resource use by women six and twelve months postpartum using a COI analysis with a bottom-up approach. Findings showed greater health resource use across all services for women with perinatal anxiety compared to those without perinatal anxiety. Women used services less at twelve months than they did at six months, mainly due to decreases in secondary service use, such as psychology and mental health services.

Subgroup analyses suggested women from ethnic minority groups with perinatal anxiety used health services less than women of white ethnicity. Further research in this area focusing on ethnic minority women would provide further insight into the health resource use of women with perinatal anxiety and how these services may be improved in a culturally and linguistically appropriate way that is cost-effective, potentially reducing the long-term cost burden and informing perinatal mental health service strategy.

### How findings fit with existing literature

This is the first comprehensive COI analysis of NHS health resource use for perinatal anxiety disorder conducted in the UK [25]. It is consistent with COI studies in the United States (US) of other maternal mental health conditions, such as postnatal depression, which show that maternal mental health conditions cost significantly more in health resource service use. For example, a 2016 US study investigated out-of-pocket expenses and insurer expenses for depressed versus non-depressed mothers. This study found that depressed mothers were more likely to incur insurer and out-of-pocket expenses and to have higher insurer expenses ($4916 vs. $3521) and out-of-pocket expenses ($786 vs. $522) [26]. Similarly, another US study found that healthcare expenditure for depressed women was significantly higher than for non-depressed women, at $1046.3 and $365.2, respectively [27]. Long-term costs of for children are also affected. Moore Simas et al., (2020) found that service use was higher for children of mothers with postnatal depression than those without depression across most services. Additionally, within the first 24 months of life, children of mothers with postnatal depression incurred 12% higher total healthcare costs than children of mothers without postnatal depression ($24,572 versus $21,946; *p* < 0.001).

### Strengths and limitations

The present study provides an overview of self-reported healthcare resource use among women six and twelve months postpartum in the UK. The COI bottom-up approach provided a comprehensive snapshot of the health resources used by women with and without perinatal anxiety within a UK health service perspective. Although the sample at both time points includes participants from ethnically diverse backgrounds, the findings may not be representative of other parts of the UK, such as Wales ​[28]. Additionally, it was not possible to include a wider societal perspective of costs in this sample due to misinterpretation by participants surrounding monthly and annual income.

Due to the high percentage of participants of white ethnicity and low percentage of those from a minority ethnic background, as per the general population of the UK, there is only a marginal difference in total mean costs within this subgroup analysis. There was a small sample size of subgroup analysis, for example, 46% missing data. Missing data can introduce bias and reduce the reliability of the findings. Therefore, the subgroup analysis should be interpreted with caution.

It is possible that perinatal anxiety not only affects healthcare costs, but also wider societal costs. For example, women with perinatal anxiety were slightly less likely to return to work 12 months postpartum than women without perinatal anxiety. Although we observed this difference, we did not collect data on potential mediating factors, such as access to childcare, transport, or other social and practical supports, which may have contributed. Future research could examine these factors in relation to both return to work and overall societal cost impact. Economic analysis in this area with a focus on wider societal costs is needed to investigate the broader economic impact of perinatal anxiety.

This analysis did not examine the overlap in the use of mental health nurses and psychologists among women with perinatal anxiety which could indicate greater severity or persistence of anxiety and may contribute to variation in individual healthcare costs. However, the main drivers of healthcare costs were inpatient stays and primary care contacts, and mental health professional use represented a smaller proportion of total costs. Future studies could explore patterns of combined mental health service use and their relationship to cost and clinical outcomes.

This analysis focused on perinatal anxiety, however, co-existing conditions such as depression or general psychological distress, which were measured in the MAP study, may also contribute to healthcare costs. Future analyses could explore the combined or interacting effects of multiple perinatal mental health conditions on health resource use and costs.

The small differences in hospital stay duration between women with and without perinatal anxiety may reflect individual variation or unmeasured comorbidities and should be interpreted with caution given the small number of admissions.

### Policy implications

Although it is estimated that 30 to 50% of women with PMH problems are identified, only 7% were referred to specialist care in the UK in 2016 [29, 30]. This coincides with this study, in which 38% of participants self-reported with perinatal anxiety. This finding has implications for health policy in which more needs to be done to ensure that women with perinatal anxiety are receiving the services they need.

Additionally, a higher proportion of women who had previously experienced psychological and mental health problems self-reported as having perinatal anxiety. Implementation of better care for perinatal women who have previously experienced psychological and mental health problems could effectively reduce the health service cost burden for perinatal anxiety.

## Conclusion

This study provides a rigorous, detailed description of the COI for perinatal anxiety to health services in the UK. Results show increased use of health services by women with perinatal anxiety at both six and twelve months postpartum. At twelve months, women with and without perinatal anxiety were using services less than they were at six months.

Findings from this study can assist future health economics research examining the cost-effectiveness of other therapeutic approaches and management strategies that could be effective for treating women with perinatal anxiety. Further studies in this area should focus on greater understanding of the health resource use of women from ethnic minority groups and how services could be improved in a culturally and linguistically appropriate way that is cost-effective, potentially reducing the long-term cost impact to health services and wider society.

## Supplementary Information

Supplementary Material 1.

## Data Availability

All the necessary data pertaining to this study can be found in the supplementary file.
