# Supplementary material for: Healthcare use and costs of perinatal anxiety: a UK NHS perspective
Source: BMC Health Serv Res. 2025 Sep 3;25:1183. doi: 10.1186/s12913-025-13318-z (PMC12406475; doi:10.1186/s12913-025-13318-z)
Supplement: Supplementary file 1 — Supplementary Material 1. [file 12913_2025_13318_MOESM1_ESM.docx]

| **Supplementary file 1****Table 1: Unit costs of primary and secondary care** | | | | | |
| --- | --- | --- | --- | --- | --- |
| **Detail** | **Level of care** | **Unit cost** |  | **Unit** | **Source/notes** |
| **Primary health care** |  |  |  |  |  |
| Community midwife | Primary | £94.00 |  | per activity | Assumed community postnatal. (NHS 2021/22, Other currencies, N01P). |
| GP | Primary | £42.00 |  | per visit | Unit cost per surgery/clinic contact lasting on average 9.22 minutes. Includes qualification costs and direct care staff costs (PSSRU, 2022, p.70). Assumed one visit was average lasting 9.22 minutes. |
| GP practice nurse | Primary | £52.00 |  | per hour | Unit cost per hour (Band 6). Includes qualification costs (PSSRU, 2022, p.68). |
| Health visitor | Primary | £116.00 |  | per activity | Assumed health visitor 1 year review. (NHS 2021/22, Other currencies, N03D). |
| **Secondary health care (free texts)** |  |  |  |  |  |
| **Other Healthcare professionals** |  |  |  |  |  |
| Allergy specialist | Secondary | £139.00 |  | per hour | Unit cost per hour including qualifications, Associated specialist, PSSRU 2022, p. 102 |
| Audiologist | Secondary | £111.11 |  | Unit cost | NHS 2021/22, Audiometry or Hearing Assessment, 4 years and under, Currency code: CA37C |
| Breastfeeding supporter | Secondary | £34.00 |  | per hour | Assumed as Clinical support worker. Unit cost per hour (Band 3). Excludes qualification costs (PSSRU, 2022, p.95). |
| CBT therapist | Secondary | £278.00 |  | unit cost | NHS 2021/22, Cognitive behavioural therapy as part of a pain management programme, Total HRGs (Total), Currency: AB11Z |
| chiropodist | Secondary | £69.00 |  | Unit cost | NHS 2021/22, Podiatrist, Tier 1, General Podiatry, Currency A09A, Other Currencies |
| Chiropractor | Secondary |  |  |  | Do not cost for NHS perspective |
| Clinical psychologist | Secondary | £64.00 |  | per hour | Unit cost per hour (Band 7). Excludes qualification costs (PSSRU, 2022, p.60). |
| Consultant medical | Secondary | £146.00 |  | per hour | Unit cost per hour (Band 6). Includes qualification costs (PSSRU, 2022, p.102). |
| Consultant psychiatrist | Secondary | £146.00 |  | per hour | Unit cost per hour (Band 6). Includes qualification costs (PSSRU, 2022, p.102). |
| Consultant surgical | Secondary | £144.00 |  | per hour | Unit cost per hour (Band 6). Includes qualification costs (PSSRU, 2022, p.102). |
| Counsellor | Secondary | £42.00 |  | per hour | Unit cost per hour (Band 5). Excludes qualification costs (PSSRU, 2022, p.60). |
| Dermatologist | Secondary | £146.00 |  | per hour | Unit cost per hour (Band 6). Consultant medical, Hospital-based doctor, Includes qualification costs (PSSRU, 2022, p.102). |
| Dietitian | Secondary | £77.00 |  | per activity | Dietitian. (NHS 2021/22, Other currencies, A03). |
| District nurse | Secondary | £53.74 |  | Unit cost | NHS 2021/22. District nurse, adult, face to face, currency N02AF |
| Doctor | Secondary | £73.00 |  | per hour | Unit cost per hour. Registrar, Hospital-based doctor, Includes qualification costs (PSSRU, 2022, p.102). |
| Gynaecologist | Secondary | £146.00 |  | per hour | Unit cost per hour (Band 6). Consultant medical, Hospital-based doctor, Includes qualification costs (PSSRU, 2022, p.102). |
| Health visitor | Secondary | £116.00 |  | per activity | Assumed health visitor 1 year review. (NHS 2021/22, Other currencies, N03D). |
| Mental nurse | Secondary | £48.00 |  | per hour | Unit cost per hour (Band 5). Includes qualification costs (PSSRU, 2022, p.98). |
| Midwife | Secondary | £48.00 |  | per hour | Assumed it costs as nurse. Unit cost per hour (Band 5). Excludes qualification costs (PSSRU, 2022, p.95). |
| Midwife consultant | Secondary | £148.00 |  | per hour | Unit cost per hour (Band 9) including qualifications. PSSRU, 2022, p.98. |
| NHS dentist | Secondary | £138.00 |  | per hour | Assumed NHS dentist performer only. Unit cost per hour of patient contact (PSSRU, 2022, p.74). |
| Nurse | Secondary | £52.00 |  | per hour | Unit cost per hour (Band 5). Qualified nurse includes qualification costs (PSSRU, 2022, p.66). |
| Nurse specialist | Secondary | £59.00 |  | per hour | Unit cost per hour (Band 6). Includes qualification costs (PSSRU, 2022, p.98). |
| Occupational therapist | Secondary | £99.00 |  | per activity | Physiotherapist, adult, one to one. (NHS 2021/22, Other currencies, A06A1). |
| Osteopath | Secondary | £41.00 |  | per visit | Assumed it costs as Physiotherapist. Unit cost per hour (Band 5). Excludes qualification costs (PSSRU, 2022, p.95). |
| Pharmacist | Secondary | £53.00 |  | per hour | Unit cost per hour (Band 6). Pharmacist, hospital-based scientific and professional staff, excludes qualification costs (PSSRU, 2022, p.102). |
| Physiotherapist | Secondary | £73.00 |  | per activity | Physiotherapist, adult, one to one. (NHS 2021/22, Other currencies, A08A1). |
| Psychiatrist | Secondary | £146.00 |  | per hour | Unit cost per hour (Band 6). Consultant psychiatry, Hospital-based doctor, Includes qualification costs (PSSRU, 2022, p.102). |
| Psychologist | Secondary | £64.00 |  | per hour | Unit cost per hour (Band 7). Clinical psychologist excludes qualification costs (PSSRU, 2022, p.95). |
| Radiologist | Secondary | £41.00 |  | per hour | Unit cost per hour (Band 5). Radiologist, hospital-based scientific and professional staff, excludes qualification costs (PSSRU, 2022, p.95). |
| Sonographer | Secondary | £73.00 |  | per hour | Unit cost per hour (Band 8a). Sonographer, hospital-based scientific and professional staff, excludes qualification costs (PSSRU, 2022, p.95). |
| Special midwife | Secondary | £106.00 |  | per hour | Unit cost per hour (Band 8c). Midwife consultant, hospital-based nurse, includes qualification costs (PSSRU, 2022, p.98). |
| Speech and language therapist | Secondary | £128.00 |  | Unit cost | Unit cost per visit. Speech and language therapist, Adult, one to one. (NHS 2021/22, Other currencies, A13A1). |
| Support worker | Secondary | £25.00 |  | per hour | Unit cost per hour, Support and outreach worker, PSSRU 2022, p.87 |
| **Hospital services (free texts)** |  |  |  |  |  |
| Accident & Emergency | Secondary | £144.00 |  | Unit cost | NHS reference costs 2021/22. (Total Outpatient Attendance) Emergency Medicine Service (service code 180) |
| Ambulance | Secondary | £268.39 |  | Unit cost | NHS reference costs 2021/22 (Ambulance) See & Treat |
| Ambulatory care | Secondary | £62.90 |  | Per visit | Unit cost per visit. Assumed Hear & Treat service. (NHS 2021/22, Currency code 1). |
| Audiology service | Secondary | £126.00 |  | per activity | Total - Audiology Service (service code 840). (NHS 2021/22, Total Outpatient Attendance). |
| Blood test | Secondary | £4.70 |  | Unit cost | NHS reference costs 2021/22 (DAPS) Phlebotomy (Currency Code DAPS08) |
| Breast clinic | Secondary | £184.00 |  | Unit cost | NHS 2021/22 Consultant led, Index CL |
| Burn clinic | Secondary | £143.00 |  | Unit cost | NHS 2021/22 Burns Care Service, Total Outpatient Attendance, Service code:161 |
| Cardio service | Secondary | £169.00 |  | Unit cost | NHS 2021/22 Cardio service, Total Outpatient Attendance, Service code:320 |
| Clinical Haematology Service | Secondary | £194.00 |  | Unit cost | NHS reference costs 2021/22. Total Outpatient Attendance - Clinical Haematology Service (303) Unit cost £194 total outpatient attendance for DVT clinic |
| Colonoscopy | Secondary | £510.00 |  | Unit cost | NHS 2021/22. Diagnostic Colonoscopy, 19 years and over (Total HRGs tab, Outpatient procedures, currency: FE32Z) |
| CT scan | Secondary | £104.97 |  | Unit Cost | NHS reference costs 2021/22. Imaging Outpatient - Computerised Tomography Scan of One Area, without Contrast, 19 years and over (IMAGOP: RD20A) |
| Day case | Secondary | £1,038.00 |  | per visit | Unit cost per visit (NHS 2021/22, Index, DC). |
| Dermatology service | Secondary | £152.00 |  | Unit cost | NHS reference costs 2021/22. Total Outpatient Attendance - Dermatology Service - Total (service code 330) |
| Diagnostic Imaging Service | Secondary | £46.00 |  | Unit cost | NHS reference costs 2021/22. Total Outpatient Attendance - Diagnostic Imaging Service (Total) Service code: 812 |
| Ear Nose throat service | Secondary | £155.00 |  | Unit cost | NHS reference costs 2021/22. Total Outpatient Attendance - Ear Nose throat Service (Total) Service code: 120. |
| Endoscopy | Secondary | £540.00 |  | Unit cost | NHS reference costs 2021/22. Total HRGs - Diagnostic Endoscopic Upper Gastrointestinal Tract Procedures, 19 years and over (Outpatient procedure) Currency: FE22Z |
| Family planning service | Secondary | £104.00 |  | Unit cost | NHS reference costs 2021/22. Total Outpatient Attendance - Family planning clinic (Total) Service code: FPC. |
| Fetal medicine service | Secondary | £201.00 |  | Unit cost | NHS reference costs 2021/22 - Total Outpatient Attendance - Fetal medicine service (Total) Service code: 505. |
| Gastroenterology service | Secondary | £149.00 |  | Unit cost | NHS 2021/22 Gastroenterology Service, Total outpatient attendance, service code: 301 |
| General dental service | Secondary | £192.00 |  | Unit cost | NHS reference costs 2021/22 - Other Currencies, General Dental Service, Contact (M01B) |
| General outpatient attendance | Secondary | £184.00 |  | Unit cost | NHS 2021/22 Consultand led, Index CL |
| General surgery service | Secondary | £161.00 |  | Unit cost | NHS reference costs 2021/22 - Total Outpatient Attendance - General surgery service (Total) Service code: 100 |
| Gynaecology service | Secondary | £181.00 |  | per visit | Unit cost per visit. (NHS 2021/22, Total outpatient attendance, 502). |
| Gynaecology Service | Secondary | £181.00 |  | Unit cost | NHS reference costs 2021/22 - Total Outpatient Attendance -Gynaecology Service (Total) Service code: 502. |
| Hepatology Service | Secondary | £192.00 |  | Unit cost | NHS reference costs 2021/22 - Total Outpatient Attendance - Hepatology Service (Total) Service code: 306. |
| Medical Assessment Unit (MAU) | Secondary | £242.00 |  | per visit | Assumed MAU is costed as same as A&E. Unit cost per visit (NHS 2021/22, Index, EC). |
| Medical Oncology Service | Secondary | £206.00 |  | Unit cost | NHS reference costs 2021/22 - Medical Oncology Service - Total (Service Code 370) 'Total Ouptaient Attendance'. |
| Midwifery service | Secondary | £120.00 |  | Unit cost | NHS reference costs 2021/22 - Total Outpatient Attendance - Midwifery service (Total) Service code: 560 |
| MRI | Secondary | £188.11 |  | Unit Cost | NHS reference costs 2021/22 - Imaging Outpatient - Magnetic Resonance Imaging Scan of One Area, without Contrast, 19 years and over (IMAGOP: RD01A) |
| Neurology Service | Secondary | £214.00 |  | Unit cost | NHS reference costs 2021/22 - Neurology Service - Total (Service Code 400) 'Total Ouptaient Attendance'. |
| NHS 111 | Secondary | £8.69 |  | per intervention | Costed as a Telephone triage - Nurse-led triage (PSSRU, 2022, p.73). |
| Occupational therapy service | Secondary | £106.00 |  | per visit | Unit cost per visit (NHS 2021/22, Total outpatient attendance, 651). |
| Ophthalmology Service | Secondary | £142.00 |  | Unit cost | NHS reference costs 2021/22 - Ophthalmology Service, Unit costs £142, Total Outpatient Attendance, Service code: 130 |
| Orthodontic Service | Secondary | £211.00 |  | Unit cost | NHS reference costs 2021/22 - Total Outpatient Attendance - Orthodontic Service (Total) Service code: 143 |
| Orthopaedic service | Secondary | £157.00 |  | Unit cost | NHS reference costs 2021/22 - Total Outpatient Attendance - Orthopaedic Service (Total) Service code: 111 |
| Out of hour | Secondary | £8.69 |  | per intervention | Costed as a Telephone triage - Nurse-led triage (PSSRU, 2022, p.73). |
| Paediatric Emergency Medicine Service | Secondary | £356.00 |  | Unit cost | NHS 2021/22 Paediatric Emergency Medicine Service, Total outpatient attendance, service code: 270 |
| Perinatal mental health service | Secondary | £180.00 |  | per visit | Unit cost per visit. (NHS 2021/22, Total outpatient attendance, 724). |
| Pharmacy service | Secondary | £53.00 |  | per hour | Costed as a Band 6 Hospital-based Pharmacist (PSSRU, 2022, p.94). |
| Physiotherapy service | Secondary | £100.00 |  | per visit | Unit cost per visit (NHS 2021/22, Total outpatient attendance, 650). |
| Rheumatology Service | Secondary | £165.00 |  | Unit cost | NHS reference costs 2021/22 - Total Outpatient Attendance - Rheumatology Service (Total) Service code: 410. |
| Triage | Secondary | £8.69 |  | per intervention | Costed as a Telephone triage - Nurse-led triage (PSSRU, 2022, p.73). |
| Ultrasound | Secondary | £77.81 |  | Unit Cost | NHS reference costs 2021/22 - Imaging Outpatient - Ultrasound Scan with duration of less than 20 minutes, without Contrast (IMAGOP: RD40Z) |
| Urgent treatment Centre | Secondary | £242.00 |  | per visit | Assumed it costed as A&E. Unit cost per visit (NHS 2021/22, Index, EC). |
| Urology Service | Secondary | £138.00 |  | Unit cost | NHS reference costs 2021/22 - Total Outpatient Attendance -Urology Service (Total) Service code: 101. |
| Vaccine | Secondary | £40.00 |  | Unit cost | NHS reference costs 2021/22 - (Other Currencies - total) Specialist Nursing, Treatment Room Nursing Services, Adult, Face to face (Currency code N27AF) |
| X-ray | Secondary | £41.41 |  | Unit cost | NHS reference costs 2021/22 - Imaging Outpatient - Plain Film (IMAGOP: PF) |

# **Table 2: Unit costs of Inpatient stay**

| **Spell cost** | **Excess bed day cost** | **Trim point** | **HRG Code** | **Sources/References** |
| --- | --- | --- | --- | --- |
| £2,879.00 | £266.00 | 14 | AA22G | Annex A NHS reference costs 2021/22 - APC & OPROC - Cerebrovascular Accident, Nervous System Infections or Encephalopathy, with CC Score 0-4 |
| £463.00 | £266.00 | 5 | AB21Z | Annex A NHS reference costs 2021/22 - APC & OPROC - Epidural or Therapeutic Spinal Puncture, for Pain Management |
| £1,947.00 | £238.00 | 11 | DZ11V | Annex A NHS reference costs 2021/22 - APC & OPROC - Lobar, Atypical or Viral Pneumonia, without Interventions, with CC Score 0-3 |
| £1,070.00 | £238.00 | 5 | DZ22Q | Annex A NHS reference costs 2021/22 - APC & OPROC - Unspecified Acute Lower Respiratory Infection without Interventions, with CC Score 0-4 |
| £4,107.00 | £238.00 | 26 | DZ25H | Annex A NHS reference costs 2021/22 - APC & OPROC - Fibrosis or Pneumoconiosis, with Intervention, with CC score 0-6 |
| £802.00 | £238.00 | 5 | DZ28B | Annex A NHS reference costs 2021/22 - APC & OPROC - Pleurisy with CC Score 0-2 |
| £853.00 | £273.00 | 5 | EB04Z | Annex A NHS reference costs 2021/22 - APC & OPROC - Hypertension (NEL) |
| £3,858.00 | £273.00 | 12 | EY23C | Standard Other Percutaneous Transluminal Repair of Acquired Defect of Heart with CC Score 0-4 |
| £2,821.00 | £269.00 | 6 | FF37D | Annex A NHS reference costs 2021/22 - APC & OPROC - Appendicectomy Procedures, 19 years and over, with CC Score 0 |
| £3,317.00 | £269.00 | 12 | FF60D | Annex A NHS reference costs 2021/22 - APC & OPROC - Complex Hernia Procedures with CC Score 0 |
| £4,624.00 | £267.00 | 13 | GA10J | Annex A NHS reference costs 2021/22 - APC & OPROC - Assumed Salpingectomy same cost as Laparoscopic Cholecystectomy, 19 years and over, with CC Score 1-3 |
| £1,398.00 | £267.00 | 8 | GC18B | Annex A NHS reference costs 2021/22 - APC & OPROC - Non-Obstructive Jaundice with CC Score 0-8 |
| £934.00 | £251.00 | 5 | KB02K | Annex A NHS reference costs 2021/22 - APC & OPROC - Diabetes with Hyperglycaemic Disorders, with CC Score 0-1 |
| £1,681.00 | £248.00 | 14 | LA04S | Annex A NHS reference costs 2021/22 - APC & OPROC - Kidney or Urinary Tract Infections, without Interventions, with CC Score 0-1 |
| £513.00 | £316.00 | 5 | SA44A | Annex A NHS reference costs 2021/22 - APC & OPROC - Single Plasma Exchange or Other Intravenous Blood Transfusion, 19 years and over |
| £1,284.00 | £247.00 | 5 | WH07G | Annex A NHS reference costs 2021/22 - APC & OPROC - Infections or Other Complications of Procedures, without Interventions, with CC Score 0-1 |
| £889.00 | £247.00 | 5 | WH08B | Annex A NHS reference costs 2021/22 - APC & OPROC - Unspecified Pain with CC Score 0 |
| £2,688.00 | £276.00 | 18 | YF04C | Annex A NHS reference costs 2021/22 - APC & OPROC - Percutaneous Single Drainage of Abdominal Abscess, with CC Score 0-1 |
| £377.00 | £276.00 | 5 | YQ51E | Annex A NHS reference costs 2021/22 - APC & OPROC - Deep Vein Thrombosis with CC Score 0-2 |
| £801.00 |  |  |  | NHS reference costs 2021/22 - Non-elective inpatient - short stay, NES |
| £1,038.00 |  |  |  | NHS reference costs 2021/22, Day case (Index DC) |
| £4,409.00 |  |  |  | NHS reference costs 2021/22, Non-elective long stay (NEL) |

*For people who did not put reasons/wards for inpatient stay (0.5%), non-elective inpatient stay (short/long) were used as a unit cost instead. Long stay was defined as length of stay was 3 or more nights.

# **Table 3: Sensitivity analysis of mean total health resource use costs by removing outliers (£, SD)**

|  | **Main analysis** | | | **Removing outliers** | | |
| --- | --- | --- | --- | --- | --- | --- |
| **Details** | 6 months postnatal | 12 months postnatal^a^ | 12 months postnatal^b^ | 6 months postnatal | 12 months postnatal^a^ | 12 months postnatal^b^ |
| **With** | £1,174 (808.11)* | £414 (537.04) | £408 (513.82) | £1,046 (591.58)** | £252 (187.31) | £265 (213.10) |
| **Without** | £1,046 (834.18)* | £267 (459.35) | £263 (441.49) | £938 (524.95)** | £203 (175.63) | £205 (202.94) |
| ^a^, women who completed 6 and 12 months | | | | | | |
| ^b^, women who completed 12 months data collection including 72 participants who completed only 12 months data collection  * 31 outliers were removed (15 and 16 outliers were removed for women with and without perinatal anxiety, respectively)  **40 outliers were removed (27 and 13 outliers were removed for women with and without perinatal anxiety, respectively) | | | | | | |

# **Table 4: Subgroup analysis** **on pregnancy history, ethnicity, miscarriage or stillbirth history, and regional home address (mean total health care costs per person at 6 and 12 months postnatal, £ (SD))**

| **Resource use category** | | **6 months** | | | | **12 months** | | | |
| --- | --- | --- | --- | --- | --- | --- | --- | --- | --- |
|  |  | n | With | n | Without | n | With | n | Without |
| Ethnicity | |  |  |  |  |  |  |  |  |
|  | White | 228 | 1,197 (836.65) | 342 | 1,084 (888.85) | 170 | £429 (570.25) | 300 | £262 (415.75) |
|  | Non-white | 26 | 981 (622.08) | 73 | 903 (611.69) | 18 | £374 (287.96) | 61 | £311 (673.80) |
| Parity |  |  |  |  |  |  |  |  |  |
|  | First child | 161 | 2,453 (1,624.53) | 250 | 2,363 (1,647.40) | 170 | 388 (546.22) | 253 | 238 (314.24) |
|  | Had previous pregnancy | 101 | 2,067 (1,466.75) | 190 | 1,990 (1,481.52) | 104 | 459 (520.81) | 195 | 305 (596.29) |
| Previous miscarriage or stillbirth | |  |  |  |  |  |  |  |  |
|  | Yes | 75 | £1,208 (775.04) | 117 | £1,022 (629.00) | 77 | £539 (782.57) | 119 | £272 (508.23) |
|  | No | 67 | £1,010 (676.69) | 124 | £897 (653.52) | 69 | £406 (403.25) | 128 | £307 (568.81) |
| Region | |  |  |  |  |  |  |  |  |
|  | North England | 52 | £1,257 (1,515.84) | 83 | £1,107 (723.74) | 41 | £455 (483.64) | 73 | £287 (248.22) |
|  | South England | 7 | £953 (713.35) | 18 | £880 (479.98) | 4 | £216 (130.79) | 15 | £250 (190.38) |
|  | London | 32 | £893 (624.97) | 81 | £1,099 (777.04) | 28 | £416 (561.60) | 72 | £233 (576.23) |
|  | Midlands | 57 | £1,031 (988.18) | 82 | £1,047 (678.42) | 47 | £468 (781.82) | 73 | £249 (266.81) |
|  | Scotland | 35 | £995 (791.07) | 59 | £912 (569.19) | 28 | £333 (364.68) | 52 | £277 (411.67) |
